# Measuring Tumor Mutational Burden (TMB) in Plasma from mCRPC Patients Using Two Commercial NGS Assays

**DOI:** 10.1038/s41598-018-37128-y

**Published:** 2019-01-14

**Authors:** Ping Qiu, Christian H. Poehlein, Matthew J. Marton, Omar F. Laterza, Diane Levitan

**Affiliations:** Merck & Co., Inc., 2000 Galloping Hill Road, Kenilworth, NJ 07033 United States of America

## Abstract

Tumor tissue mutational burden (TMB) has emerged as a promising predictive biomarker for immune checkpoint therapy. Measuring TMB from circulating tumor DNA (ctDNA) found in plasma is attractive in tissue-constrained indications. We compared the performance of two plasma-based commercial TMB assays including the effect of two different collection methods. Our findings suggest that the two plasma based TMB assays are highly correlated and they are also both correlated with a tissue-based TMB assay for relatively high TMB samples. The two collection methods are also found to be very comparable. Plasma-based TMB assays may be mature enough to be clinically useful in mCRPC and potentially other indications.

## Introduction

Liquid biopsies are more convenient, less expensive and less risky to the patient than standard tumor biopsy^1^. Tumor tissue mutational burden (TMB) has emerged as a promising predictive biomarker for immune checkpoint therapy. Measuring TMB from circulating tumor DNA (ctDNA) found in plasma is attractive^2^ in indications such as metastatic castration resistance prostate cancer (mCRPC), where obtaining tissue can be challenging.

Chalmers *et al*.^3^ demonstrated that TMB can be accurately measured by sequencing targeted gene panels but that accuracy is compromised when the sequenced genome region (bait size) is less than 0.5 MB. The Guardant Health (GH) Omni (500 genes, 2.1 MB) and Foundation Medicine (FMI) bTMB (394 genes, 1.14 MB) panels are plasma-based NGS assays containing sufficiently large bait sizes to measure TMB across a broad range of TMB values. Poor concordance on mutation detection between two commercial vendors was reported by Torga and Pienta previously^4^. We compared the performance of TMB determination of the two plasma assays in this study, including the effect of two different collection methods.

## Methods

Replicate sets of plasma samples from 20 mCRPC patients (2 ml each; Streck tube protocol with double centrifugation^5^) were sent to GH and FMI for analysis. In addition, one set of samples from the same 20 subjects collected in EDTA tubes^6^ (spun once, stored at −70 °C, spun again prior to NGS assay at GH) were also sent to GH to investigate the impact of different pre-analytical collection methods in determination of TMB. Matching formalin-fixed paraffin-embedded (FFPE) tissue from each subject (collected within twelve months of plasma collection) was analyzed by WES at Personal Genome Diagnostics. All methods were carried out in accordance with relevant guidelines and regulations. All experimental protocols were approved by Merck Ethics Review Committees. Informed consent was obtained from all subjects or, if subjects are under 18, from a parent and/or legal guardian.

## Results

**Figure 1:**
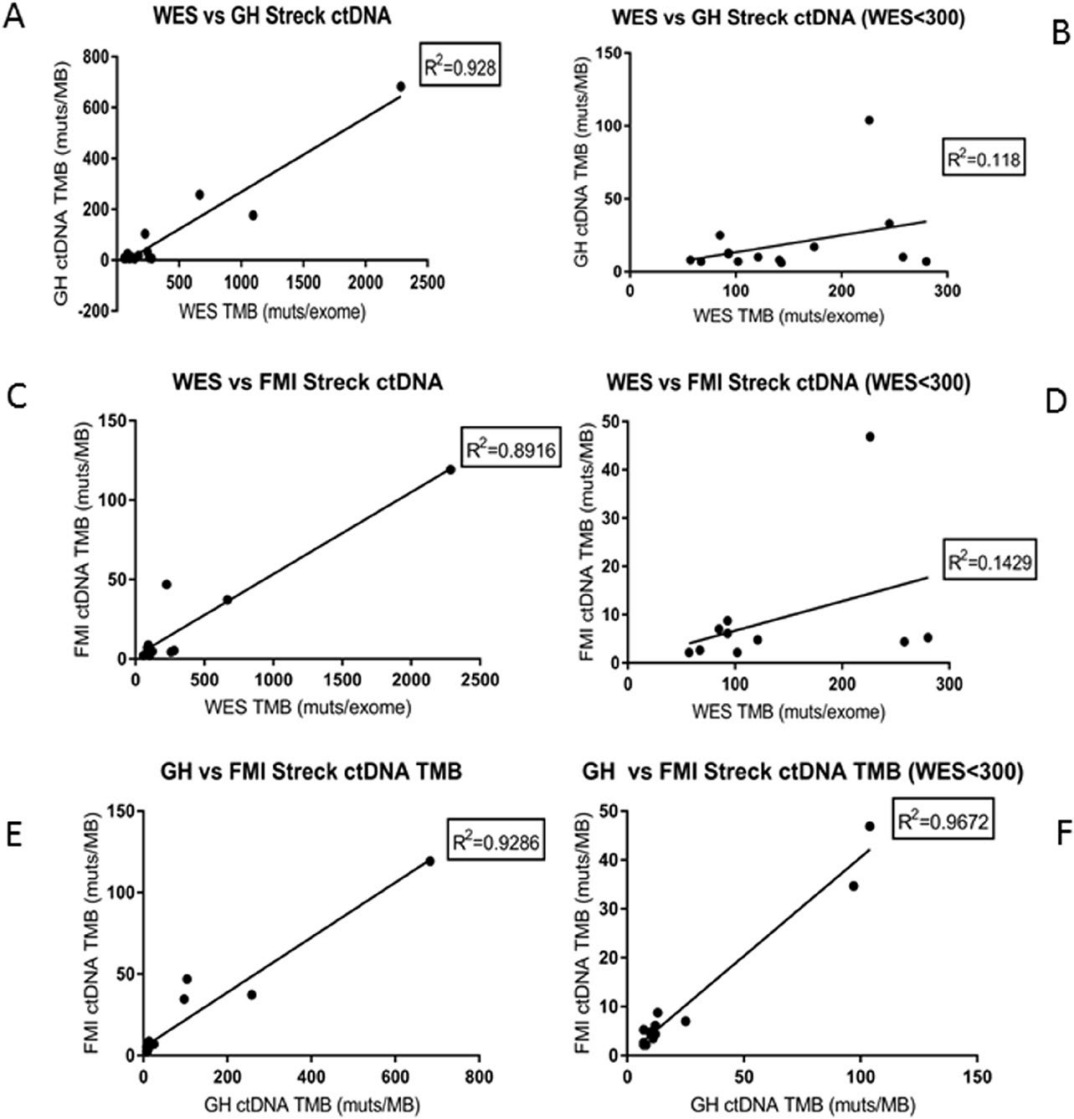
Comparison of TMB measured by WES (muts/exome) in tissue biopsies and TMB measured from ctDNA (muts/MB) obtained from Streck tubes by GuardantHealth Omni and Foundation Medicine bTMB assays.

TMB measured by the FMI bTMB and GH Omni assays is summarized in Fig. 1. A high correlation (R^2^ ≈ 0.9) between blood TMB assays (muts/MB) and tissue WES (muts/exome) was observed in general (Fig. 1A,C), although correlation for low/medium tissue TMB samples was not as high as high TMB samples (R^2^ ≈ 0.1; Fig. 1B,D). A similar low correlation between targeted panel NGS and WES at low TMB level was also demonstrated in a tissue based assay by Buchhalter *et al*. using TCGA data^7^. However, the two plasma based TMB assays are highly correlated (R^2^ > 0.9) even for biopsy samples with low/medium TMB (Fig. 1E,F), which suggests that the low concordance between tissue and blood for low TMB samples may be more likely to be biological rather than technical. The GH Omni panel reports all four types of mutations in addition to TMB, while the FMI bTMB assay is validated to report blood TMB only. Therefore, it was not possible to evaluate concordance of target gene mutation detection between these two panels in this study.

**Figure 2:**
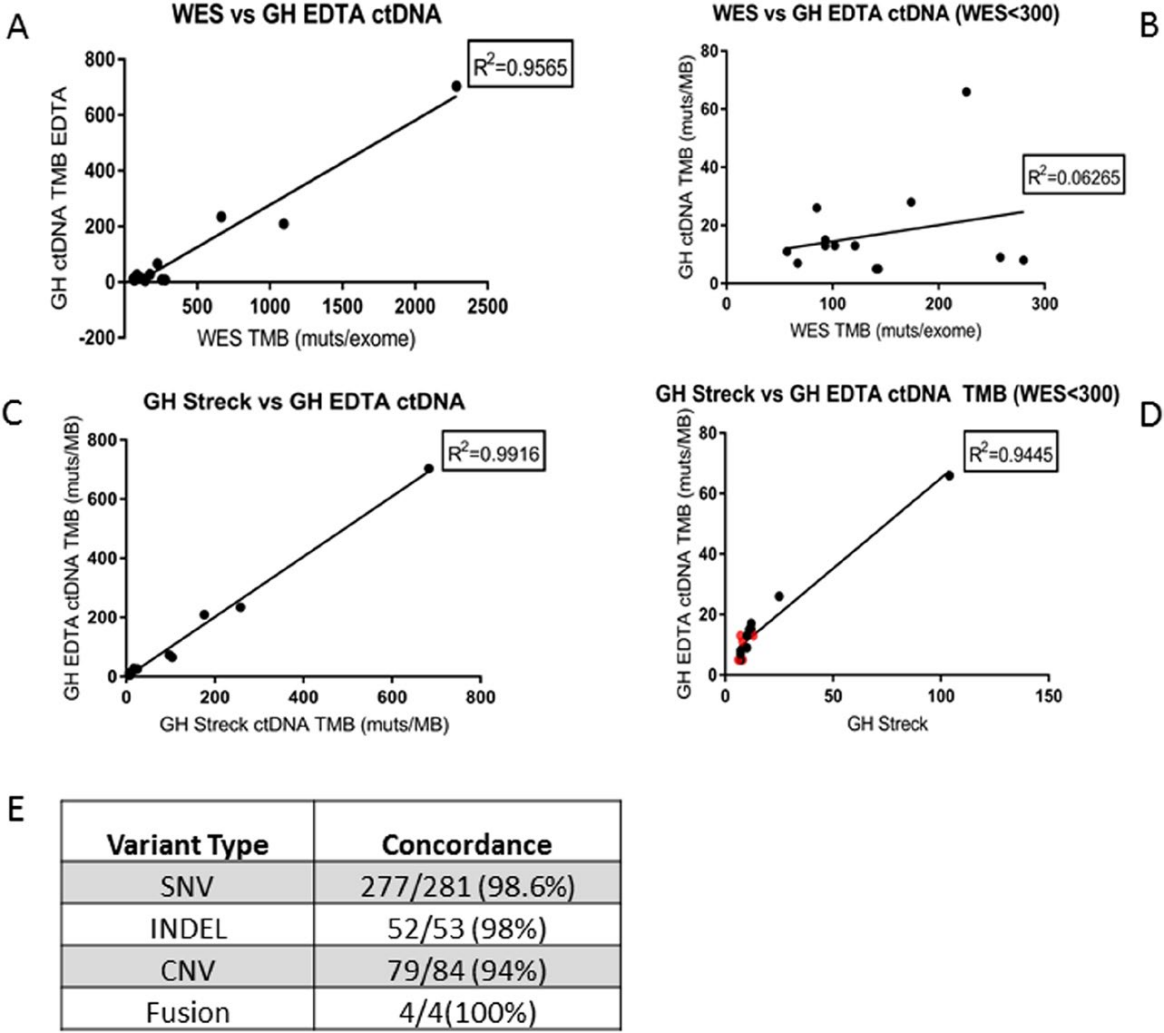
Comparison of TMB measured from plasma collected either by Streck protocol or the EDTA protocol using GH Omni assay. Samples with unmatched collection time (less than one month difference) between Streck and EDTA are highlighted in red in D.

It is worth noting that the absolute values of TMB (muts/MB) from the two plasma assays are different. This is likely due to difference in sequencing depth, TMB algorithm implemented and allele frequency cutoff used in the two assays. Fig. 2 shows that the two collection methods have a minimal impact on TMB assessment. Four types of mutations (SNP, Indel, CNV, Fusion) detected from the two collection methods are highly concordant (Fig. 2E). The cell free DNA yield from the two collection methods are very comparable (data not shown) which suggests that the freeze-thaw impact is minimal as long as a first spin is done before freezing.

## Discussion

Kuderer *et al*.^8^ reported a large discordance in driver gene mutation detection between tissue and plasma ctDNA based assays. In this study, although mutation level concordance was not compared, good correlation between tissue and plasma ctDNA TMB is observed for both assays for high TMB samples. However, correlation is compromised between tissue and plasma ctDNA based assays for low/medium TMB samples. Given the biological difference between plasma and tissue and the magnitude of sequencing depth differences between these two assays, a good correlation between tissue WES and plasma assays may not necessarily be expected. This study suggests the clinical validity of plasma TMB and tissue TMB in immunotherapy be evaluated independently. Another important finding of this study is that EDTA plasma may be a suitable specimen for the determination of TMB. This is important information for investigators interested in performing retrospective analysis in studies in which plasma samples may not have been collected in Streck tubes. Although additional studies with larger sample size and clinical outcome data are warranted to further substantiate these preliminary findings and evaluate the clinical utility of plasma TMB, these preliminary results are promising and suggest plasma-based TMB assays may be mature enough to be clinically useful in mCRPC and potentially other indications.

## References

[1] Merker JD (2018). Circulating Tumor DNA Analysis in Patients With Cancer: American Society of Clinical Oncology and College of American Pathologists Joint Review. J Clin Oncol..

[2] Kim ST (2018). Comprehensive molecular characterization of clinical responses to PD-1 inhibition in metastatic gastric cancer. Nat Med..

[3] Chalmers ZR (2017). Analysis of 100,000 human cancer genomes reveals the landscape of tumor mutational burden. Genome Med..

[4] Torga G, Pienta KJ (2018). Patient-Paired Sample Congruence Between 2 Commercial Liquid Biopsy Tests. JAMA. Oncol..

[5] Sacher AG (2016). Prospective Validation of Rapid Plasma Genotyping for the Detection of EGFR and KRAS Mutations in Advanced Lung Cancer JAMA. Oncol..

[6] Sherwood, J. L *et al*. Optimised Pre-Analytical Methods Improve KRAS Mutation Detection in Circulating Tumour DNA (ctDNA) from Patients with Non-Small Cell Lung Cancer (NSCLC). *Plos One*, 10.1371/journal.pone.0150197 (2016). 10.1371/journal.pone.0150197 PMC4769175 26918901

[7] Buchhalter, I. *et al*. Size Matters: Dissecting Key Parameters for Panel-Based Tumor Mutational Burden (TMB) Analysis. *Int J Cancer*, 10.1002/ijc.31878 (2018). 10.1002/ijc.31878 30238975

[8] Kuderer NM (2017). Comparison of 2 Commercially Available Next-Generation Sequencing Platforms in Oncology. JAMA. Oncol..

